# Molecular Dynamics Simulation of Calcium-Silicate-Hydrate for Nano-Engineered Cement Composites—A Review

**DOI:** 10.3390/nano10112158

**Published:** 2020-10-29

**Authors:** Byoung Hooi Cho, Wonseok Chung, Boo Hyun Nam

**Affiliations:** 1 Department of Civil, Environmental and Construction Engineering, University of Central Florida, 12800 Pegasus Drive, Suite 211, Orlando, FL 32816, USA; byoungcho@ucf.edu; 2 Department of Civil Engineering, Kyung Hee University, 1732 Deogyeong-daero, Giheung-gu, Yongin-si 17104, Korea

**Keywords:** molecular dynamics, calcium-silicate-hydrate, nano-engineered cement materials, carbon-based nanomaterials, cement–polymer nanocomposites

## Abstract

With the continuous research efforts, sophisticated predictive molecular dynamics (MD) models for C-S-H have been developed, and the application of MD simulation has been expanded from fundamental understanding of C-S-H to nano-engineered cement composites. This paper comprehensively reviewed the current state of MD simulation on calcium-silicate-hydrate (C-S-H) and its diverse applications to nano-engineered cement composites, including carbon-based nanomaterials (i.e., carbon nanotube, graphene, graphene oxide), reinforced cement, cement–polymer nanocomposites (with an application on 3D printing concrete), and chemical additives for improving environmental resistance. In conclusion, the MD method could not only compute but also visualize the nanoscale behaviors of cement hydrates and other ingredients in the cement matrix; thus, fundamental properties of C-S-H structure and its interaction with nanoparticles can be well understood. As a result, the MD enabled us to identify and evaluate the performance of new advanced nano-engineered cement composites.

## 1. Introduction

Historically, cementitious materials have been used as one of the most common and popular construction materials. The hydrated cement is composed of nanostructured multiple composite phases that include an amorphous phase, nano-/micro-size crystals, and bound water. Calcium-silicate-hydrate (C-S-H) plays a pivotal role in controlling mechanical, chemical, and transport properties of cement composites and their engineering performance as well [1]. Therefore, in order to improve and customize the macroscopic properties of cementitious materials, understanding of the structure of C-S-H at nano-/micro- level is prerequisite [2] because chemical process at molecular level eventually affects engineering performance of the composites in the bulk scale [1,3,4,5].

Nano-engineering in cementitious materials encompasses alteration and modification of cement hydrates for enhancing and modifying properties and performance in macro scale [1,6]. In addition, it also deals with characterization and prediction techniques via atomic- or molecular-level modeling for better understanding of how chemical interactions correlate with the macro-level behaviors [7,8]. Via nano-engineering processes such as nano-particles, nano-reinforcements, and chemical admixtures, mechanical and durability properties and degradation processes of cement composites could be effectively controlled and enhanced; thus, novel and smart multi-functions can be incorporated into the cement composites [9]. Fundamental knowledge in the field of the nano-engineered cement composites has been advanced with high-resolution characterization tools [9]; however, there are still many challenges in understanding material behaviors at the atomic/molecular level, solving test repeatability, and realizing cost–benefit issues, etc. [9,10].

Molecular dynamics (MD) simulation is a powerful computational method that models physical movements of atoms and molecules accounting for potential energy in a given position and then numerically computes the atomic/molecular forces based on Newton’s classical mechanics [1]. The MD simulation quantitatively determines statistical properties of multi-body systems because it models a large size of the molecular system with longer simulation durations for the required level of accuracy [11,12,13,14]. By this reason, the computational MD has been applied to cement composites to study mechanical behaviors and properties of C-S-H and other applications such as carbon-based nanomaterials, polymer–cement nanocomposites, and chemical treatments for the modification of material’s properties, etc. [15,16,17]. There are still needs and opportunities for the MD in cement composites because of diverse molecular structures of cement hydrates and introduction of numerous nano-admixtures into the cement matrix.

This paper provides a comprehensive review on MD analyses on cement composites. Fundamentals of C-S-H including formation, classifications, and molecular modeling were also first reviewed. Thereafter, MD studies on C-S-H structures are reviewed, focusing on C-S-H MD modeling, water dynamics characteristics in pore structures, and mechanical properties of the C-S-H in nanoscale. Lastly, the recent applications of MD to nano-engineered cement composites were reviewed.

## 2. Background on C-S-H

### 2.1. Formation and Classification of C-S-H

Tricalcium silicate (alite: C_3_S) and dicalcium silicate (belite: C_2_S) because constitute about 50~70% and 15~30% of Portland Cement (PC) by mass, respectively [18,19]. C_3_S dominates early hydration process of PC, which controls early-age properties of cement and concrete [20]. During that time, a large amount of calcium–silicate–hydrate (C-S-H) gel is formed by the alite hydration, whereas C_2_S hydration governs later composition of C-S-H [21]. A study on the early hydration process of alite shows the reaction begins immediately upon contacting the water. The initial reaction is a complex heterogeneous process that are congruent dissolution and incongruent dissolution with a formation of a silica-rich layer on the surface of C_3_S [22]. This alite–water reaction forms C-S-H and calcium hydroxide (CH) following the relationship by the schematic Equation (1). The hydration of C_2_S occurs similarly to the case of C_3_S and can be expressed by Equation (2). The rate of hydration is generally lower than alite. In addition, the slow process of C_2_S due to different structures of C_3_S results in low concentration of calcium ions; thus, CH crystals are generally large but the C-S-H form is similar [23]. In this reaction, roughly three Ca-O and two Si-O bonds are broken, including six O-H bonds and eventually forms C-S-H gel and portlandite (Ca(OH)_2_). The energy required for the bond formation of C-S-H and portlandite is 138 kJ/mol less than the energy released due to the bond breaking of the reactants mentioned in the above reaction. As a note, it is known that some of the added C_3_S in PC triggers an accelerated hydration process of C_2_S, as the nucleation of CH could be accelerated and thus the C_2_S dissolution process is also accelerated [22].

C_3_S + (3 − x + y) H → C_x_SH_y_ + (3 − x) CH (1)

C_2_S + (2 − x + y) H → C_x_SH_y_ + (2 − x) CH (2)

It is important to note that the formation, composition, and structure of C-S-H can be influenced even by a slight variation of w/c, mix proportion, humidity, the degree of hydration, and curing temperature [21]. Those factors contribute the ratio of calcium to silicon (Ca/Si) [24,25,26] and the variability of the C-S-H structure comes from the varied Ca/Si ratio ranged from 0.7–2.3 [10,27]. The Ca/Si ratio may increase under higher hydration and better curing conditions, but the use of supplementary cementitious materials (SCMs) may decrease the ratio. Lower Ca/Si ratios may increase the length of silicate chains and the interlayer distance, resulting in changes of C-S-H structures [26]. Generally, C-S-H was firstly classified into two categories as tobermorite-like C-S-H(I) for Ca/Si < 1.5 and jennite-like C-S-H (II) for Ca/Si > 1.5 [28]. The C-S-H(I) was additionally divided into C-S-H(α) for Ca/Si < 1.0 and C-S-H (β) for 1 < Ca/Si < 1.5 [29]. Nowadays, depending on this ratio, C-S-H can be depicted as tobermorite, jennite, metajennite, and many other formations shown in Table 1 in the next section.

It was observed that the type of C-S-H largely depends on hydration times; for instance, low-density C-S-H is rapidly formed at the surface of C_3_S in the early stage of hydration [21,30]. As the hydration process continues, a group of C-S-H having higher density is subsequently formed, which mainly contributes to the development of strength. Powers and Brownyard firstly denoted the physical characterization of C-S-H at multiscale in the 1940s and 1950s [31,32]. They suggested inside and outside hydrated products at the cement clinker particles (e.g., C_3_S and C_2_S), which were denoted as inner (Ip) and outer (Op) of C-S-H by Taplin [33]. Diamond (1976) observed four different types of C-S-H morphologies including three types of Op-C-S-H and one type of Ip-C-S-H [34]. They are classified as Type I: fibrous particles look like partly rolled sheets, Type II: reticular network/interlocking structure but not in C3S or C2S pastes, Type III: “relatively nondescript” or “equant grain morphology”, and Type IV: inner product in older pastes. Richardson and Groves investigated the formation and classification of C-S-H with transmission electron microscopy (TEM) and then adopted the terminologies of OP and IP [35,36]. Later, Jennings and Tennis have classified the C-S-H in density perspective by modeling colloidal particles that pack into two separate arrangements: high density (HD) and low density (LD) products [37,38]. They showed that the LD products are formed during the first or second day of hydration but those are more deformed during the drying stage due to its open structure than HD products [39]. Nicoleau described the formation of C-S-H being the outcome of cohesive forces during the hydration process, and its physical transformation could trigger coagulation and eventually form structures [40]. Bonding characteristics of the C-S-H formation were also investigated. The high-density C-S-H is a layered structure composed of calcium silicate sheets randomly connected by strong iono-covalent bonds [41].

### 2.2. Structural Models of C-S-H

**Figure 1:**
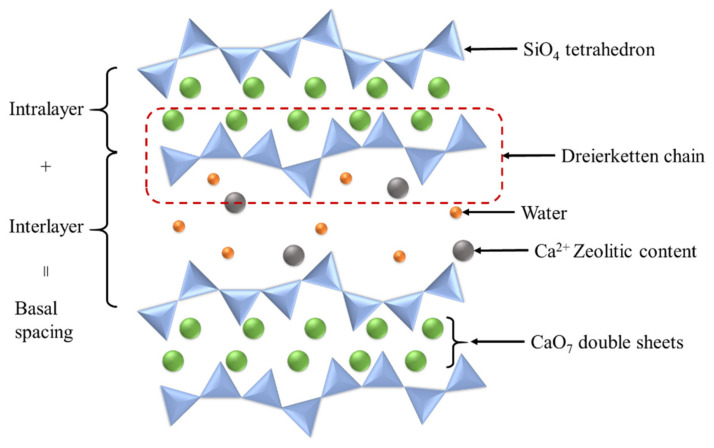
Schematic structure of crystalline C-S-H.

Modeling the C-S-H structures needs to address the variation of chemical composition and its distribution in a cement matrix because engineering performance of cement hydrates is highly affected by structures and chemical compositions of C-S-H determined by w/c ratio and curing conditions. Therefore, specifically, the model should be able to account for: (1) Ca/Si ratio of C-S-H ranged from 0.7 to 2.3 [42], (2) compositional heterogeneity of C-S-H, and (3) age of hydration process [43,44]. In an early stage of C-S-H study, Taylor (1986) announced that C-S-H appears to be a disordered layer structure composed of structurally imperfect jennite (Ca_9_Si_6_O_32_H_22_) and others similarly related to 14 Å tobermorite (Ca_5_Si_6_O_26_H_18_) [45]. Tobermorite and jennite are layered structures that are calcium sheets flanked on both side by linear silicate chains called “dreierketten chain” [46,47]. Figure 1 shows a schematic diagram of C-S-H crystalline structure. The chains are composed in a repeating manner at three SiO_4_ tetrahedra intervals. Two adjacent tetrahedrons are coordinated to the Ca^2+^s of the layer, and the third tetrahedron bridges two consecutive dimers, which is called bridging tetrahedra. Two oxygens from non-bridging tetrahedra are coordinated with Ca^2+^s in tobermorite [48,49], whereas one oxygen from the non-bridging tetrahedra is coordinated to the Ca^2+^ on the sheet and the other oxygen is provided by hydroxide ions [50,51]. Taylor described several formulas based on Ca/Si ratios: Ca_4_H_4_Si_6_O_18_·8H_2_O (Ca/Si = 0.66) and Ca_5_H_2_Si_6_O_18_·8H_2_O (Ca/Si = 0.83) for tobermorite; and Ca_5_H_2_Si_4_O_16_·8H_2_O (Ca/Si = 1.25), Ca_8_H_4_Si_6_O_18_(OH)_8_·6H_2_O (Ca/Si = 1.33), Ca_9_H_2_Si_6_O_18_(OH)_8_·6H_2_O (Ca/Si = 1.5), and Ca_9_H_2_Si_4_O_16_(OH)_8_·6H_2_O (Ca/Si = 2.2) for jennite [45]. In the early research period of Taylor, he proposed that both tobermorite-like and jennite-like structures may simultaneously exist at the early age of hydration, and the distribution could finally merge into a jennite-type structure having an intermediate composition by interacting the regions of low and high Ca/Si ratio as time passes. Thereafter, however, he suggested that the tobermorite-like and jennite-like regions could be hardly determined and could merge into each other within individual layers [52]. Unlike Taylor’s model, Cong and Kirkpatrick reported that the jennite-like C-S-H is rarely formed based on experimental studies, and they proposed tobermorite-like C-S-H model when Ca/Si < 1.5 [52,53]. There is a difference between the two models in that Tayler focused on the case of Ca/Si > 1.5 whereas Cong and Kirkpatrick focused on Ca/Si < 1.5. Nonetheless, both models involve quite disordered structures. Nonat and Lecoq later elaborated the model associated with the evolution of C-S-H in accordance with the Ca/Si ratio of 0.66~2.0 [29]. In their model, the main differences from the Taylor’s and Cong and Kirkpatrick’s models are that C-S-H structures are not necessarily to be disordered structure and the layers do not contain jennite-like regions. The X-ray diffraction (XRD) analysis of C-S-H showed similar patterns with the tobermorite over the wide range of Ca/Si ratios.

**Figure 2:**
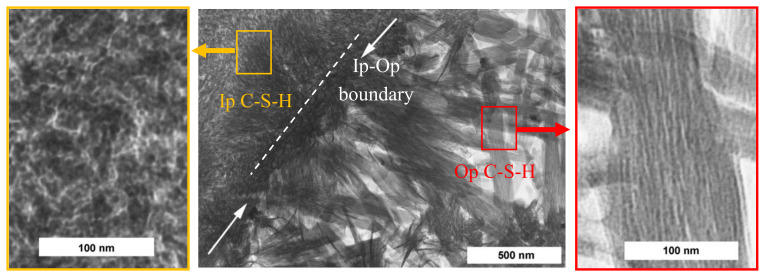
TEM image showing Ip and Op C-S-H present in a hydrated C_3_S paste. Reproduced from [54], with permission from Elsevier, 2004.

Figure 2 shows TEM images of Ip and Op C-S-H structures. The white arrows indicate the Ip–Op boundary of a hydrated C3S paste; the Ip region is in the upper left side and Op region is in the lower right side in the Figure 2, and zoom-in images of Ip C-S-H and Op C-S-H are also depicted, respectively. The Ip C-S-H appears to consist of aggregates of small globular particles being 4–6 nm in diameter whereas, the Op C-S-H looks like a bundle of fiber, about 100 nm wide, consisted of a large number of long thin particles aligned along its length.

Selected studies of C-S-H structures and the corresponding models proposed by various researchers are summarized in Table 1. It is noticed that the layer structure is the most widely adopted model in which 11 Å and 14 Å tobermorite and jennite structures are used to model the structure of C-S-H. Accordingly, these C-S-H structures have been mainly adapted for the MD simulation of C-S-H to be presented in following sections.

## 3. Molecular Dynamics Simulation of C-S-H

### 3.1. MD Models of C-S-H

**Figure 3:**
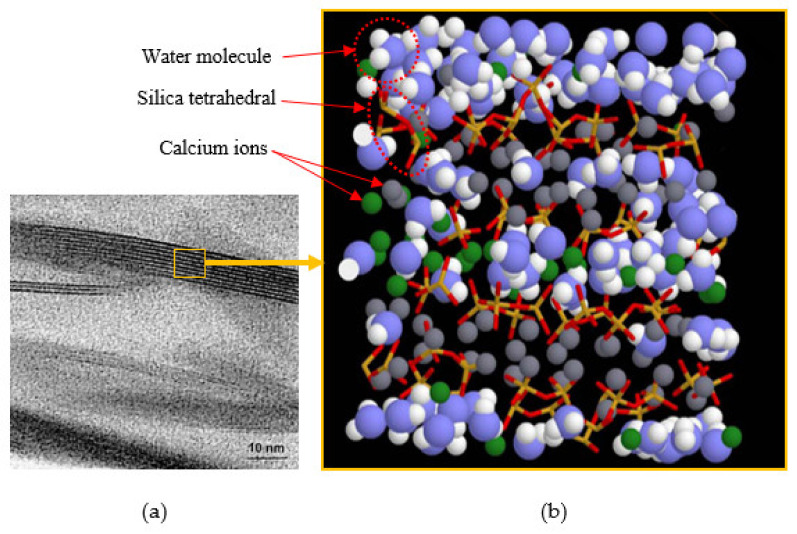
C-S-H structure and model: (**a**) TEM image of C-S-H clusters [70], (**b**) molecular model of C-S-H ((CaO)_1.65_(SiO_2_)(H_2_O)_1.75_). The blue and white spheres are oxygen and hydrogen atoms of water molecules, respectively; the green and gray spheres are inter- and intra-layer calcium ions, respectively; yellow and red sticks represent silicon and oxygen atoms in silica tetrahedral [69].

Since the 2000s, MD simulation of C-S-H has been explored and helped to investigate the structures, behaviors, and diverse properties of hydrated cement matrix at molecular level [2]. MD theoretically computes a set of molecular orbital phase spaces where each molecule follows Newton’s laws of motion [68]. In other words, the MD simulation method calculate a typical trajectory of the molecular system based on the fact that the total energy is constant. As this is a deterministic algorithm, initial conditions use random velocities, and it is usually known as an initial configuration from Monte Carlo simulations. Accordingly, understanding properties and behaviors of hydrated cement in small scale will help understand the macroscopic behaviors. Pellenq et al. (2009) first suggested the C-S-H molecular model by MD method [69]. The model was developed based on the mean value of 1.7 for Ca/Si ratio repeating 14 Å tobermorite crystalline structure, which was suggested by Richardson [36]. Figure 3 shows the TEM image of C-S-H clusters [70] and the corresponding MD model by Pellenq et al. [69] The blue and white spheres are oxygen and hydrogen atoms of water molecules, respectively. The green and gray spheres are inter- and intra-layer calcium ions, respectively. Yellow and red sticks represent silicon and oxygen atoms in silica tetrahedral. The overall chemical composition of the computational model of hydrated C-S-H is (CaO)_1.65_(SiO_2_)(H_2_O)_1.75_. The C-S-H model is based on a bottom-up atomistic simulation approach (i.e., atomic to composite scale) which considers only the chemical specificity of the system as a major constraint. This C-S-H pattern that involves the interaction of CaO, SiO_2_ and H_2_O molecules allows the distributions of short silica chains such as monomers, dimers and pentamers, and thus, it could provide more realistic Ca/Si ratio and density values calculated by grand canonical Monte Carlo simulation of moisture adsorption at 300 K.

Table 2 summarizes the selected studies of MD simulation on C-S-H structures and experimental methods for validation. The table also summarizes the applications of MD analyses to C-S-H, looking into the structures of C-S-H, diffusion coefficients of water, density, elastic modulus, tensile/compressive/shear strength, chloride diffusion, and the most stable structures corresponding to minimum energy. One of the most important parameters in the simulation of C-S-H is Ca/Si ratio. Researchers used different Ca/Si ratio to simulate the closest structure to the C-S-H gel, which ranged from 0.66 to 2.0, however, the typical ratio of Ca/Si in hydrated cement composite is considered as 1.7. 9 Å, 11 Å, and 14 Å tobermorite layered C-S-H structures have been mainly modeled. Tobermorite structures could have several modifications under different levels of hydration and degree of cross-linking of silicate chains between two adjacent layers, and this could form different levels of separation of the interlayer. According to the spacing due to the separation, the structures are named by the 9 Å, 11 Å, and 14 Å tobermorite. On the other hand, only a few studies attempted to model amorphous C-S-H clusters [68]. MD simulation software (i.e., GULP, LAMMPS, and TREMOLO) involves NVT (Number of atoms, Volume and Temperature are constant) or NPT (Number of atoms, Pressure and Temperature constant). Appropriate potentials are used to simulate the real-life interactions (i.e., 2-body or 3-body) among the molecules. Reactive force field ReaxFF is useful for capturing bond breaking and reforming by considering fracture [71], electrostatic equilibrium, and water dissociation [72], and thus, it is widely adapted for simulating systems having dynamically changing bond topologies [73]. Whereas the ReaxFF reproduce the energy evolution due to the bonding and dissociating atoms, empirical force fields such as CLAYFF and CSHFF predefine the connections between atoms at a fixed state [74]. CLAYFF force field combines coulombic, van der Waals, bonded stretch and angle bend potentials altogether. On the other hand, CSHFF is more popular for investigating water dynamics and cohesion between C-S-H grains [75]. Accordingly, the empirical force fields have been employed for studying the nano-engineered cementitious materials and its applications such as nanomaterial-reinforced cement and polymer modified cement, which to be presented in the following sections.

### 3.2. Water Dynamics in C-S-H

**Figure 4:**
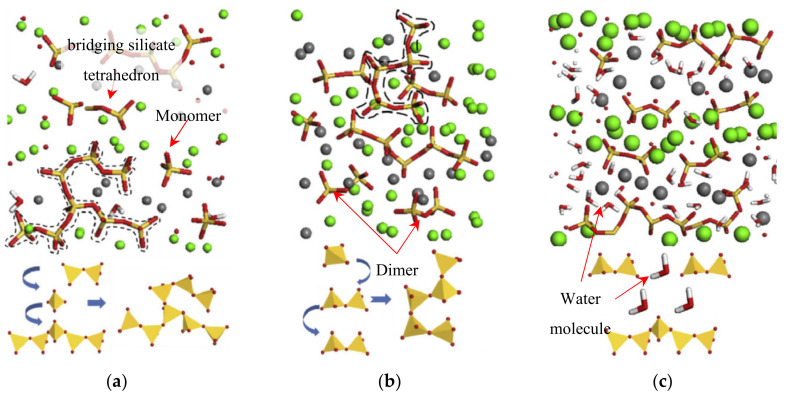
Morphology change of silicate chain: (**a**) dry condition—bridging silicate tetrahedrons connect to surrounding monomers; (**b**) water/Ca = 0.3—two dimers and one monomer develop a long silicate chain; and (**c**) saturated condition—dimer structures grow orderly along one direction. Reproduced from [101], with permission from Elsevier, 2014.

The pore system having a wide range of size distribution from nano- to micro- meters significantly affect physical and chemical properties of hydrated cement composites such as strength, shrinkage, creep, and chemical reactivity [77]. Since 1970s, extensive studies about water interactions which include water bound into cement paste, “glassy water” interacted in gel pores, and unbound water in larger capillary pores in the near surface of cement hydrates have been made; for instance, dynamics of unbound water, physically bound and chemically bound water under variable conditions [88,89,90,91,92,93,94,95,96]. Hou et al. (2014, 2015) simulated C-S-H nanostructure under varied moisture conditions (from dry to saturated conditions) based on twelve (12) C-S-H gel structures with water/Ca ratios from 0 to 0.95. Specifically, they investigated the chemical bonds in the C-S-H structure using “CSHFF force field” [97,98,99,100]. Figure 4 illustrates the morphology change of silicate chains in C-S-H models under different water contents. In the dry state, the bridging silicate tetrahedron is connected to the surrounding monomers and adjacent tetrahedrons. When the water/Ca ratio is 0.3, two dimers and one monomer can be linked together and form a longer silicate chain. In the saturated state, the dimer structure develops orderly, and the water molecules block the silica chains from connecting to each other.

The behavior of confined water in nanometer scale greatly differs than that of bulk-scale water. The viscosity of the nanoconfined water (≤ 1 nm separation) is seven orders of magnitude higher than the viscosity of bulk water at room temperature [102]. Solid surfaces can perturb the confined water up to several molecular diameters [77]. Kalinichev et al. (2007) and Youssef et al. (2011) simulated water dynamics in the pore systems of cement matrix. Kalinichev’s MD analyses illustrate that (1) water molecules develop a three-dimensional H-bond network and (2) the structure of the H-bond network can be influenced by substrate structures (e.g., C-S-H structures and/or charge distribution) [77]. In addition, Youssef’s research demonstrates that nano-pore space has hydrophilic characteristic due to non-bridging oxygen atoms on the disordered silicate chains acting as H-bond acceptor sites [103]. In their studies, the models were intentionally designed to simulate the solid-fluid interaction that is a strong attractive forces between water molecules and C-S-H chains, and used a “CLAYFF forced field” method to generate energetics between the solid-fluid interfaces that do not require the prior definition of chemical bonds. Since the disordered systems such as hydrous minerals, which is composed of a large number of atoms, could be kept relatively small and simple by modeling the number of interactions in the absence of defined chemical bonds, the O-H of H_2_O, the OH- group on the solid surface, and the bond at the aqueous oxyanion were only defined in the CLAYFF [77]. Moreover, they found that the diffusion coefficients of water molecules are much higher near the surface of solid than in the confined channels. However, the both values are much lower than the diffusivities of bulk water, which were validated using NMR technique. Consequently, it was concluded that the water at the surface of tobermorite and jennite is highly structured with reflecting the structure, composition, and charge distribution of the underlying C-S-H substrate. In these ways, MD methods offer significant potential for modeling fluid-solid interface and their interaction in cement systems.

### 3.3. Nanoscale Mechanical Properties and Performance of C-S-H

Understanding properties of C-S-H can help predict mechanical and chemical properties of hydrated cement materials, thus findings can be reflected on the design of cement/concrete composites. Manzano et al. (2007) [104] simulated different crystalline C-S-H models that have diverse Ca/Si ratios, and subsequently computed diverse elastic properties such as bulk (K), shear (G), and Young’s Modulus (E). The results showed that the values of modulus slightly decreased when Ca/Si ratio of C-S-H increased and also when more water content was added. Moreover, the study also showed that mechanical properties of C-S-H with dimer or pentamer silicate chains were lower than those with infinite silicate chains. MD studies computing elastic properties of C-S-H have been widely performed under diverse force field types. It is shown that CLAYFF generally estimates higher modulus values than other types such as COMPASSFF, COMPASSIIFF, or Universal, but estimates Poisson’s ratio in the middle range [105]. Table 3 summarizes the computed elastic properties of diverse C-S-H structures; however, nanoindentation tests show much lower values. The test results report bulk modulus (K) of 15–18 GPa, shear modulus (G) of 9.7 GPa, and Young’s modulus (E) of 18–30 GPa [65,106,107,108]. In other words, the computed moduli of C-S-H gel overestimates about 3–5 times larger than those of experimental test. 

**Figure 5:**
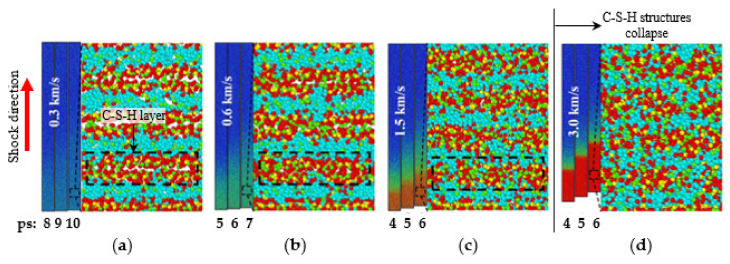
Illustration of the dynamic deformation of C-S-H under different particle velocities (*v_p_*): (**a**) *v_p_* = 0.3 km/s, (**b**) *v_p_* = 0.6 km/s, (**c**) *v_p_* = 1.5 km/s, and (**d**) *v_p_* = 3.0 km/s [118].

Murray et al. (2010) [78] studied tensile and compressive strength of the C-S-H structure from the uniaxial stress–strain data computed by MD. The results showed that the strength values of C-S-H in nanoscale is about three times of maximum compressive and tensile strength of hydrated cement at bulk scale. In their MD simulation, the tensile strength appeared 23% of the compressive strength. This study also addressed that attractive electrostatic forces and silicate bond (O-Si-O), which resist tensile stresses greater than 600 MPa, play a crucial role in determining the strength of hydrated cement, and the breakage of silicate chains is the primary cause of reduced tensile strength of C-S-H structure due to less O-Si-O bonding. The application area of MD simulation methods was also expanded as such from the conventional analysis of static performance and mechanical properties of C-S-H phases to investigate dynamic behavior characteristics such as propagation of stress waves under shock loading. For instance, the pattern of crack propagation may change as the strain rate increases due to higher shock wave; a higher stress level is required for the failure of specimen, and the energy absorption capacity increases [115,116,117]. Lin et al. (2018) investigated the propagation of stress waves in the C-S-H under the compressive shock loading by selecting the particle velocity of 0.2–3.0 km/s for the wave generation using MD simulations [118]. The elastic limit was calculated as 7.5 GPa, and the results also presented that 0.5 km/s is the critical particle velocity to transit the C-S-H structure from an elastic state to a plastic state. If the particle velocity is larger than 2.0 km/s, the C-S-H structure is collapsed due to the shock wave. Figure 5 illustrates the dynamic-deformation process of C-S-H structures under different particle velocities simulated by MD method. The black-dotted box in the figure indicates C-S-H layer, and the plastic deformation is related to the densification of the C-S-H layers. Figure 5b shows the C-S-H layer begins to compact, indicating that the system transits from elastic to plastic at the particle velocity of 0.6 km/s. Figure 5d demonstrates that the system lost the layered C-S-H morphology at the particle velocity of 3.0 km/s, and it is considered as structure collapse.

**Figure 6:**
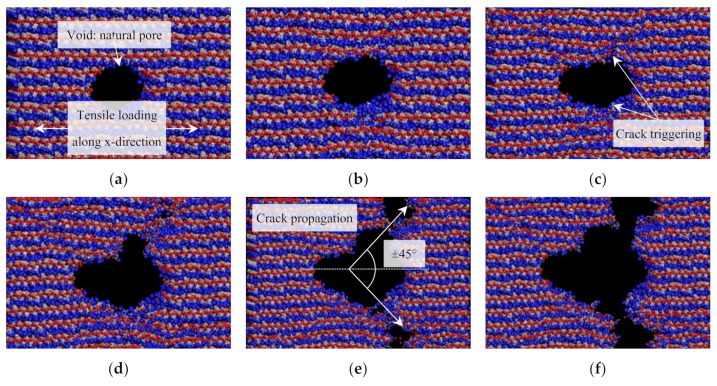
MD simulation of crack evolution of C-S-H gel under tensile load along the *x*-direction: (**a**) *ε* = 0, (**b**) *ε* = 0.08, (**c**) *ε* = 0.16, (**d**) *ε* = 0.2, (**e**) *ε* = 0.3, and (**f**) *ε* = 0.4 [100].

Crack propagation mechanism in C-S-H gel is also studied via the MD method. Hou et al. (2014) reported that the layered C-S-H gel shows dual types of crack growth natures under loading conditions [100]. In the *x–y* plane, since the ionic-covalent bonds of Si-O and Ca-O are stable, cracks are hardly coalesced in this direction, which may slow down the crack propagation and thus lead to more ductile characteristic. On the other hand, in the *z*-direction, cracks could be propagated in the interlayer region more frequently because of higher potential to break the H-bonds network under loading, and this could be exhibited as a brittle behavior in macroscale. They also simulated the development of crack under the tensile loading process in which a void is present at the center of C-S-H. The void could be a natural pore having diameter of 0–50 Å generated within hydration process rather than defects. Figure 6 demonstrates the crack evolution of C-S-H gel under the tensile load along the *x*-direction simulated through MD method. Figure 6a–f represent the strain at 0, 0.08, 0.16, 0.2, 0.3, and 0.4 Å/Å, respectively. As shown in Figure 6c, the crack is triggered from the boundary of central void by dissociating Si-O and Ca-O bonds as strain increases from 0.08 to 0.16. Consequently, it could be identified in Figure 6e that the cracks coalesce with small cracks and propagates to the direction of around ±45° from the *x*-axis. This MD simulation provides considerable insight regarding the reduced plastic deformation during the cracking process of C-S-H gel. First of all, water molecules hinder the interflow of Ca and Si between adjacent calcium silicate sheets, thus weakens the plastic deformation of the C–S–H gel. Next, the water molecules instantly spread out to bond to the non-bridging oxygen site in the damaged region, and thus obstruct bridging by the interlayer calcium, resulting in the plastic deformation due to the fact that Ca, Si, and O diffusion may be reduced [100].

## 4. MD Simulations on Nano-Engineered Cement Materials

### 4.1. Carbon-Based Nanomaterials

#### 4.1.1. Carbon-Based Nanomaterials-Reinforced Cement Composite

**Figure 7:**
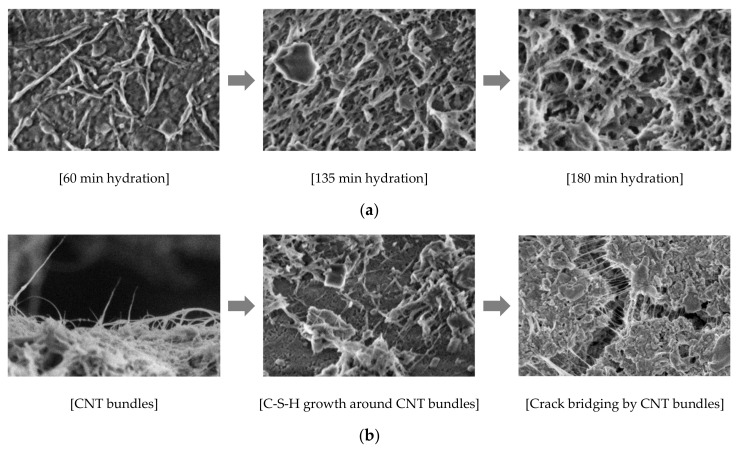
(**a**) Nucleation effect of CNT: increased thickness of C-S-H on CNT bundles as hydration proceeds. Reproduced from [130], with permission from Wiley Online Library, 2009; and (**b**) bridging effect of CNT bundles at cracks in C-S-H [132].

It has been reported that carbon-based nanomaterials (e.g., carbon nanotubes/nanofibers (CNTs/CNFs) and graphene/graphene oxide (GO)) could enhance mechanical properties such as Young’s modulus (E) and tensile strength (*f_t_*); for instance, E = 10–950 GPa and *f_t_* = 11–150 GPa for CNT, E = 25–600 GPa and *f_t_* = 7–30 GPa for CNF, E = 1.5–2.8 TPa and *f_t_* = 130–195 GPa for pristine graphene, and E = 180–230 GPa and *f_t_* = 0.11–24 GPa for GO [119,120,121,122,123,124,125,126,127]. Cwirzen et al. indicated that adding CNTs to the cement paste improved the workability and increased the compressive strength up to 50% [128]. In addition, pristine CNTs could improve the flexural and compressive strength of the cement composites about 10–20% [129]. One of the reasons for the improvement of mechanical strengths is a nucleation effect of CNT allowing the growth of C-S-H gel in the early hydration process reported by Makar and Chan [130] (Figure 7a). They confirmed the CNT’s nucleation effect by measuring the decrement of Ca(OH)_2_ during the hydration process for the CNT-containing cement paste. A “bridging effect” of CNTs in the cement matrix is considered as another mechanism for the strength enhancement, which could increase the tensile strength of C-S-H as they can bridge the micro and nanocracks in the C-S-H (Figure 7b) [131,132,133], and hence can be termed as “bridging material” hereafter. Numerical investigation of Eftekhari et al. [134,135] presented that the addition of CNTs to the cement mixtures could significantly increase the fracture energy and tensile strength of the CNT-reinforced cement and delay the crack propagation. Manzur [136] addressed that the strength improvement could be adjusted by the CNT’s specific surface area based on experimental results that the compressive strength of CNT–cement composite increases at the larger specific surface area of CNT, and this could be supported by that COOH or C-OH groups on the surface of CNT allow a possible interaction with C-S-H gel [137].

Graphene oxide (GO) has been considered as a viable alternative for reinforcing cementitious matrices with better economic advantages than CNT [138,139,140]. The interfacial strength of pristine graphene with –OH, –COOH and –NH_2_ functionalized groups in the cement composites have been studied by AlKhateb et al., and the results presented that the electrostatic forces of the functional groups play a roles in determining the strength of GO-reinforced cement composites [141]. In addition, it is reported that the functionalized surface of GO may act as a seeding material accelerating the hydration process, thus contributing to increase the amount of C-S-H gel in the matrix [142,143,144,145,146]. Consequently, previous studies have shown the positive effects of GO on mechanical properties of the cementitious composites. In summary, for the cement paste and mortar, 0.02–0.08% of GO by weight of cement increased the compressive strength by 29–46% and flexural strength by 26–61% [147,148,149,150,151,152,153,154,155]. Moreover, Lv et al. performed advanced mechanical testing and reported the GO nanosheets significantly affect to reduce brittleness and enhance toughness of the cement based materials [156]. Effective reinforcement potential was also investigated in nanoscale showing mitigation of crack propagation and lead to less sudden failure [157,158,159], and enhancement of Young’s modulus of the GO-reinforced cement composites was evaluated [160,161]. The accelerated C-S-H formation due to GOs may also reduce porosity and hinder the ingress of water and chloride ions, which subsequently improve its durability [122,162]. For the unhardened fresh state of the GO-mixed cement mixtures, it is generally known that the GOs noticeably reduce the fluidity and viscosity of cement paste and mortar mixtures, and this is understood by the fast early cement hydration process due to the functionalized surface of GO [121,163].

In recent, a low-cost GO, edge oxidized graphene oxide (EOGO), has been introduced [164] and relevant investigations on both rheological and mechanical aspects (including long-term fatigue behavior) of GO–cement and concrete composites have been performed; the result demonstrated that the EOGO could improve not only static compressive/flexural strength but also flexural fatigue characteristics (i.e., fatigue life and ductility) [165,166,167]. The results also indicated the plastic strain of the GO-reinforced cement mortar increased under flexural cyclic loading condition, and this was interpreted as the EOGOs helped prevent or delay internal damage propagation in the material. In addition, EOGO was also mixed with steel fibers in cement concrete mixtures, and the results showed the 0.1% GO by weight of cement improve the energy absorption capability of about 30% in the range of elastic behavior under the static flexural loading condition. It is assumed that the GOs extend the range of elasticity and “interact” with steel fibers in cement paste [168,169].

#### 4.1.2. MD Simulation on CNT/Graphene/GO-Reinforced C-S-H

**Figure 8:**
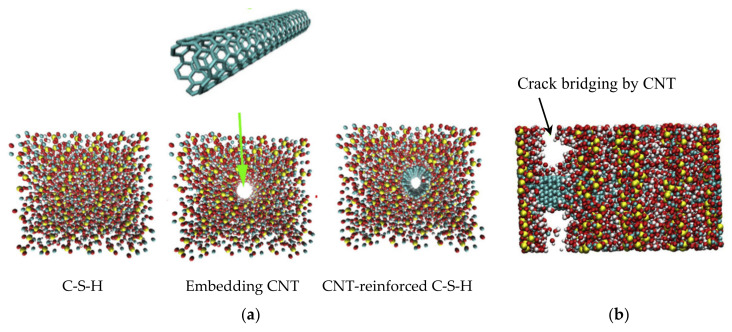
Schematic representation of (**a**) construction of CNT-reinforced C–S–H and (**b**) CNT’s crack bridging behavior. Reproduced from [170], with permission from Elsevier, 2016.

Eftekhari and Mohammadi (2016) studied the mechanical properties of CNT-reinforced C-S-H by MD simulation (see Figure 8) [170]. The MD model is firstly processed by that a hollow cylinder was placed in the C-S-H structure and expanded up to the CNT’s diameter using “fix indent cylinder” function of LAMMPS. This pushes C-S-H atoms back without the breakage of their bonds. CNT was embedded in the hollow hole, and the C-S-H and CNT was then linked by Lennard-Jones (L-J) potential. After the system equilibrium, the interlayer space between C-S-H and carbon atoms by the L-J interaction was set as about 2.7 A. The simulation results confirm that the CNT embedment enhances diverse mechanical properties of C-S-H. The tensile strength of the CNT-reinforced C-S-H is substantially improved along the direction of CNTs. Especially the CNT increased the tensile strength along the Z direction (perpendicular to the silicate layer) up to 6 GPa. The results also demonstrated that the CNTs play a role in efficiently bridging (“bridge effect”) cracks of C-S-H (see Figure 8b). In addition, the pullout simulation of CNT estimated the bilinear force-displacement response model, which can provide a viable mechanism to be used for the understanding of crack propagation and bridging effects at macro-scale.

The study of MD simulation on graphene–cement composite was pioneered by Sanchez and Zhang [171], investigating the molecular-scale energetic, structural, and dynamic properties of the interface between the surface-functionalized graphitic structures and C-S-H. In this study, six types of functional groups on the surface of carbon nanosheet were considered: hydroxyl (OH), carboxyl (COOH), carboxylate (COO−), carbonyl (CO), and amine (NH_2_) groups. As a C-S-H model, 9 Å tobermorite structure was used. The simulation results demonstrate that electrostatic forces play a pivotal role in interfacial interactions between the functionalized carbon sheet and C-S-H (i.e., 9 Å tobermorite). It was also identified that the polarity of functionalized groups could be used as an indicator of affinity for C-S-H. Later on, Alkhateb et al. investigated the interfacial strength between C-S-H and graphene nanoplates functionalized by different chemical groups, and the results presented that the functional groups such as OH, NH_2_, and COOH on the graphene nanoparticles enhance the interfacial strength of 13.5 GPa, 6.1 GPa, and 11.8 GPa, respectively, depending on the electrostatic forces of the functional groups, compared to that of the unfunctionalized graphene showing 1.2 GPa [141]. Another study investigating tensile and shear properties of 14 Å tobermorite reinforced with a pure graphene sheet (with no functional groups) [172] indicates that the graphene sheet contributes to the improvement of *XY*-plane tensile strength of 180–360% and shear strength of 90–225% and also increases stiffness and toughness of the graphene-reinforced tobermorite structures. In the meantime, water interface enhances intermolecular friction forces between graphene and C-S-H, resulting in improved tensile strength and toughness but lower shear toughness than that of the dried surface condition.

**Figure 9:**
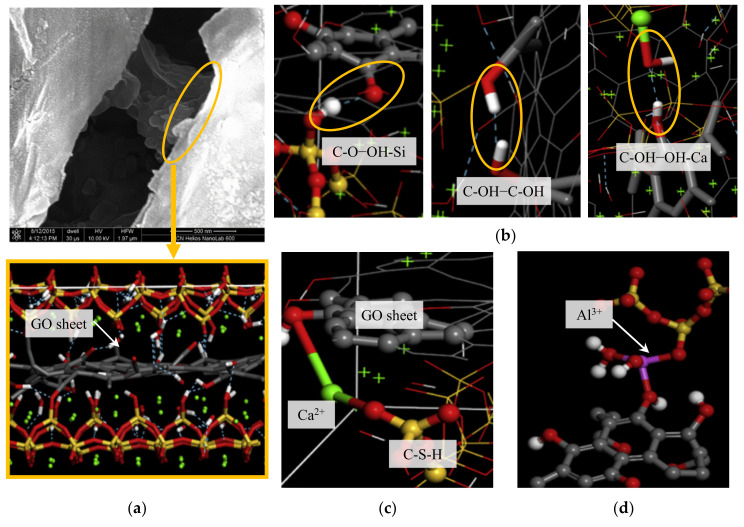
MD simulation of (**a**) GO-reinforced C-S-H, (**b**) three types of H-bonds, (**c**) O-Ca-O connection, and (**d**) O-Al-O connection [173].

MDs has been used to investigate mechanical behavior of GO-reinforced cement composites, particularly looking into the interaction between the GOs and C-S-H phases. Hou et al. [173] investigated the effects of GO on the microstructure of hydrated cement and the corresponding mechanism. The test results showed that the GO increased flexural strength about 11.62%. The simulation studies indicated GOs enable greater hydration and cause nano-filling and crack-bringing effects (see Figure 9a). The functional hydroxyl groups in GOs provide non-bridging oxygen (NBO) sites that accept three types of hydrogen-bonds (i.e., C-O–OH-Si, C-OH–C-OH, and C-OH–OH-Ca) of interlayer water molecules in the C-S-H; thus, this may change polarity of GO surface and enhances the bonding with neighboring phases (see Figure 9b). Furthermore, Ca^2+^ and Al^3+^ ions near the surface of C-S-H help bridge the GO-silicate chains, thus a length of the silicate chain is increased (see Figure 9c,d). As a result, the defective GO structure is healed. It was also addressed that the aluminate-silicate chains, calcium ions, and functional hydroxyl groups in the cement matrix could establish stabilized connections between C-S-H and GOs. Kai et al., (2019) investigated chemical reactions, mechanical behaviors, and interfacial sliding of C-S-H and GO via MD simulation [174]. The simulation showed that chemical reactions such as turning epoxides into carbonyls and hydroxyls and deprotonating or detaching hydroxyls occur at the interface between GO and C-S-H matrices, resulting in high interfacial interaction energy and mechanical interlocking. Subsequently, mechanical test simulations showed that Young’s modulus and strength of C-S-H are enhanced by 31.6–52.6% and 17.5–23.3%, respectively. Hou et al., (2018) explained more in-depth strengthening mechanisms of C-S-H and GO in molecular level [175]. This study investigated the structure, reactivity and interfacial bonding of C-S-H and GO, and the results showed that most COOH groups in GO nanosheet were de-protonated to COO- groups, which forms stable COO-Ca bonds with neighboring Ca ions. The de-protonated COO- groups could also generate H bonds from Si-OH in the C-S-H gel, which improves the interfacial connection. Moreover, uniaxial tensile testing simulation demonstrated that C-S-H reinforced with GO-COOH and GO-OH had increased interfacial cohesive strength and ductility.

In addition, Yang et al. reported the decrement of failure strength of C-S-H and GO composite under moisture condition [176]. The study investigated the effect of water molecules on the bonding in the interlayer region between C-S-H and GO nanosheet functionalized by deprotonated carboxyl (COO), hydroxyl (COH), epoxy (COC) groups. The results indicate that the interlayer bonds are weakened by water molecules as the moisture content increases, resulting in decreased tensile strength of the GO/C-S-H composite.

### 4.2. Cement–Polymer Nanocomposite

**Figure 10:**
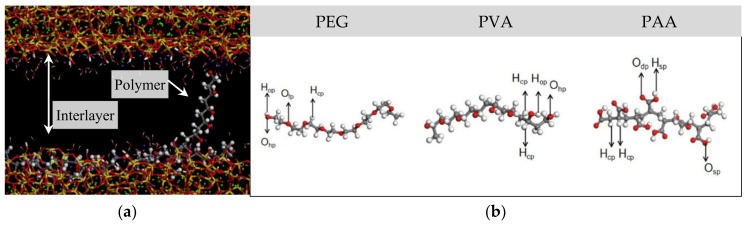
MD simulation of cement–polymer composite; (**a**) polymer chains in interlayer of C-S-H under tensile process, and (**b**) structures of polymers (PEG, PVA, PAA) with functional groups connecting to C-S-H. Reproduced from [181], with permission from Elsevier, 2019.

Polymers are widely used in cementitious materials to alter and/or improve the structures of cement matrix; thus improve mechanical and durability performance of the composites under severe environment [177,178,179,180]; thus, MDs have been used to understand chemical interactions between diverse types of polymers and cement hydrates. Hou et al., (2019) investigated the interfacial interaction between C-S-H and polymers, looking into structure, dynamics, energetics, and mechanical properties of the cement–polymer nanocomposite [181]. Polyethylene glycol (PEG), polyvinyl alcohol (PVA), and polyacrylic acid (PAA) were selected and simulated, and mechanical properties were computed for those polymer- C-S-H nanocomposites. In the MD model (see Figure 10a,b), H_op_, H_cp_, and H_sp_ denote hydrogen atoms in hydroxyl groups, connected to carbon atoms, and in –COOH carboxyl groups, respectively. O_hp_, O_fp_, O_dp_, and O_sp_ mean oxygen atoms in hydroxyl groups, in –CH_2_–, double-bonded in –COOH carboxyl groups, and single-bonded in the carboxyl groups, respectively. The simulation results show the Ca^2+^ near the surface of C-S-H connects the functional groups in the polymers to oxygen in the silicate chains by forming O_s_-Ca-O_p_ bond (where, O_s_ and O_p_ denote oxygen in silicate and polymer, respectively). In addition to the ionic bonding, functional groups in the polymers (i.e., bridging oxygen (C-O-C) in the PEG, hydroxyl (C-OH) in the PVA and carboxyl groups (-COOH) in the PAA) supply oxygen sites for the formation of H-bonds, which also enhances the connectivity of the interlayer region in C-S-H. Moreover, the computation results show that introducing polymers to C-S-H improves the H-bonds in the interface and heals defective silicate chains, resulting in delaying the propagation of crack under loading and ultimately enhancing cohesive strength and ductility of the C-S-H gels. Table 4 summarizes the results of MD simulations for the scenarios of PEG, PVA, and PAA. The PAA the most largely enhances the Young’s modulus, tensile strength, and failure strain of C-S-H to 22.3%, 19.2%, and 66.7%, respectively, and the PVA and PEG followed. This result corresponds to the rank of interfacial binding energy (E), which follows the order of E(PAA) > E(PVA) > E(PEG).

**Figure 11:**
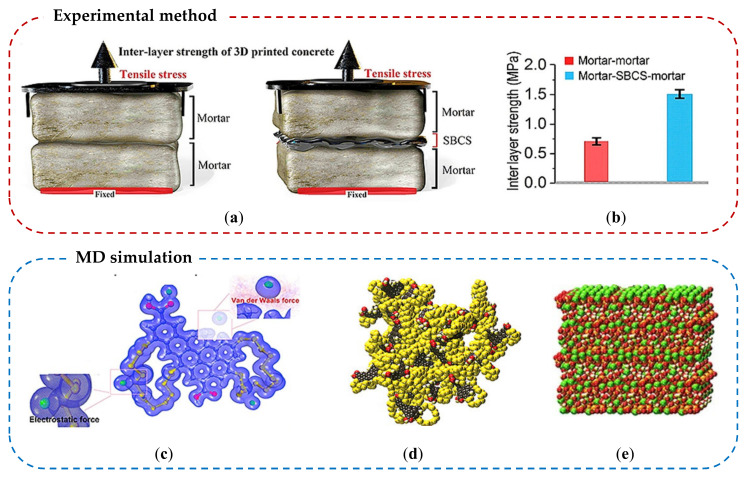
Schematic program of C-S-H/SBCS composite study; (**a**) experimental outline for tensile strength test, (**b**) interlayer strength of mortar-mortar and mortar-SBCS-mortar systems, (**c**) absorption process of Ca^2+^ on the SBC polymer, (**d**) SBC polymer MD model, and (**e**) C-S-H MD model. Reproduced from [183], with permission from Elsevier, 2019.

Polymer-modified cement composite significantly improves adhesion in the interface between layers, which is a good alternative for 3D printing concrete industry. The benefits of 3D cement/concrete printing include energy-saving and material-efficient construction methods. Wang et al., (2020) used the MD and examined the interfacial mechanical properties of polymer-modified mortar layers that is used for 3D printing [182]. In this study, epoxy resin and chloroprene latex were adopted for enhancing the interlayer bonding. Direct tensile and shear strengths of the polymer-modified cement paste were evaluated by both experimental and MD numerical methods. The results show that weak interlaminar bonding derived from water molecules is offset and can be overcome by the electrostatic interaction (Coulomb force) between the epoxy resin and Ca^2+^ from C-S-H. Consequently, enhancement of 219.55% and 201.41% of tensile and shear strengths were reported, respectively. Hosseini et al., (2019) introduced a new polymer-sand composites for enhancing the interface bonding of 3D printed cement layers [183]. They synthesized sulfur-black carbon (SBC) polymer as a binder and then mixed sulfur-black carbon-sand (SBCS) mortar for the interlayer bonding. This polymer–cement composite was evaluated by both experimental and MD methods. The results demonstrated that the CSH-CSH is mainly bonded by Van der Waals forces in the interlayer region but the CSH- SBC (mortar-SBCS-mortar) is bonded by electrostatic forces between Ca^2+^ from C-S-H and the negatively charged SBC polymer. This different chemical bonding mechanism explains that the interlayer bonding strength of CSH-SBC is enhanced by more than 100% than that of CSH-CSH. A graphical summary of experimental and MD simulation studies performed by Hosseini et al. is presented in Figure 11. It is important to note that the MD method may help find out a practical feasible material solution to advance 3D concrete printing technology.

### 4.3. Chloride Ion Binding on Cement Hydrates

**Figure 12:**
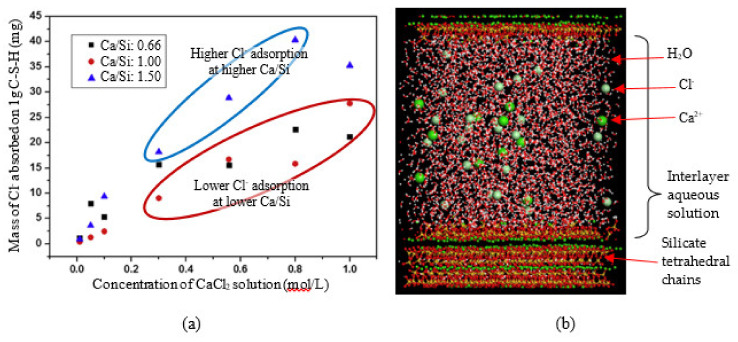
(**a**) Experimental result of Cl^−^ adsorption under different Ca/Si ratio of C-S-H, and (**b**) MD simulation of tobermorite with aqueous solutions constituted of water molecules, chloride and calcium ions; chloride ions can be bound in the interlayer of C-S-H, and the binding capacity is affected by Ca/Si ratio. Reproduced from [184], with permission from Elsevier, 2018.

Chloride intrusion in concrete material is one of the primary deterioration mechanisms that result in corrosion of reinforcement steels in concrete infrastructure. If not well attended and maintained, it may lead to catastrophic failure. C-S-H and CH possess a diverse microstructural array which allows to compose a favorable pore networks for chloride ions to intrude concrete structures [18,19], and surface physico-chemical characteristics of these cement hydrates could potentially interact with chloride ions [79]. Pan et al., (2010) [79] simulated the chloride transport phenomenon through portlandite, tobermorite, and jennite channels filled with water molecules. The simulation found that the adsorption forces from solid surface could significantly reduce the movement rate of chloride ions in the near-surface domains. It was concluded that although portlandite strongly adsorbed the chloride ions through H-bonding, the C-S-H phases did not show noteworthy binding phenomena of the chloride ions due to lower oxygen atoms. Meanwhile, Zhou et al., (2018) investigated the chloride-ion adsorption capability of the C-S-H phases at variable Ca/Si ratios [184]. They reported that higher Ca/Si condition can provide more favorable environment for binding more chloride ions. Two potential mechanisms are addressed; (1) due to the H-bond network with –OH groups, water molecules may accumulate near the surface of C-S-H, which is beneficial for the Cl^−^ adsorption, and (2) Ca^2+^ ions are stably present in the interface layer, which can also promote the Cl^−^ adsorption. Figure 12a shows experimental result of Cl^−^ adsorption under different Ca/Si ratio of C-S-H, and Figure 12b illustrates the MD simulation of tobermorite model with aqueous solutions constituted of water molecules, chloride and calcium ions developed in the study [184]. The number of water molecules was determined based on a standard density of water (1 g/cm^3^), and the concentration of chloride was selected as 0.44 mol/L that is the upper limit for the pore solution. This MD simulation results may be applied to provide a foundation to develop chemical additives and processing technology to slower the transport rate and adsorption of chlorides in reinforced concrete structures exposed to the marine environment by increasing Ca/Si ratio of the concrete mixture for the improvement of design.

## 5. Conclusions and Recommendations

This paper presents a comprehensive review on MD modeling on C-S-H and its applications in the nano-engineered cement composites. The MD is a promising tool for exploring new nano-additives because it can investigate and visualize fundamentals in a molecular level, which is difficult to be characterized by experimental methods. For instance, the MD can visually show the “bridging” effect of carbon-based nanomaterial (CNT, GO) and compute the bonding with C-S-H while experimental methods provide indirectly supporting results. The negative effect of water on the interlayer bonding between GO and C-S-H could be also investigated by the MD. For the cement–polymer nanocomposite, the MD enabled to identify the types of linking mechanisms between C-S-H and polymers; thus, computing the interfacial binding energy of polymers (i.e., PAA > PVA > PEG) was possible, which is matched well with the experimental result. The MD technique was even applied to the area of 3D-printed concrete research, and the simulation results well represented the experimental results. Lastly, the MD simulation could be effectively used in designing and assessing behaviors of chemical additives and treatment agents for the durability enhancement of cement composites under environmentally harsh conditions such as coastal areas. As nanomaterials and nanotechnology is becoming more popular and common in cement/concrete industry, the emergence of more efficient computational methods and tools capable of simulating at nanoscale are inevitable; therefore, the MD method will be an effective tool.

## Figures and Tables

**Table 1:** Analysis models describing structure of C-S-H proposed by various researchers.

Type of C-S-H Structure	Model Type				
	Layer	Colloid	Crystal	Atomic	Chain
9 Å tobermorite	[55]	-	-	-	-
11 Å tobermorite	[55,56,57,58]	-	-	-	[48]
14 Å tobermorite	[53,55,56,59,60,61,62]	-	[63]	[45]	-
Jennite	[53,54,60,61,62]	-	-	[45]	-
LD C-S-H	-	[39,64,65,66]	-	-	-
HD C-S-H	-	[65,66]	-	-	-
Op C-S-H	[54]	-	-	-	-
Hillebrandite (Ca_2_SiO_3_(OH)_2_)	-	-	[67]	-	-

**Table 2:** Summary of MD analyses on C-S-H.

Author (Year) [Ref.]	C-S-H Model	Simulation Package	Experimental Validation	Temp. (K)	# of Atoms	Ensemble Used	Energy Potential Used	Calculated Items
Faucon et al. (1997) [58]	Tobermorite 11 Å Ca/Si = 0.66 and 0.83	-	^29^Si-MAS NMR	800	2500	-	-	Structural reorganization due to cationic substitution in C-S-H
Dolado et al. (2007) [76]	Ca/Si = 0.7, 1.0, 1.4, 2.0	TREMOLO	^29^Si-MAS NMR	300–1800	6304–7448	NVT	Custom	Density
Kalinichev et al. (2007) [77]	Tobermorite 9 Å (Merlino’s model)	-	^1^H NMR	-	3646	NPT, NVT	CLAYFF	Diffusion coefficients of H_2_O molecules, Behavior of water in C-S-H and near the interface
Pellenq et al. (2008) [41]	Tobermorite	GULP, CRYSTAL	AFM	310	-	NVT	Empirical/transferable interatomic potential	Mean square displacement (MSD), self-diffusion
Pellenq (2009) [69]	Tobermorite 11 Å	GULP	SANS, NMR	300	-	NVT	-	Moduli, plane stress, strength
Murray et al. (2010) [78]	Tobermorite 9 Å (Hamid’s model)	LAMMPS	-	300	-	NPT	FF (Buckingham, Coulomb and Stillinger-Weber)	Tensile/compressive strength and elastic modulus of C-S-H
Pan (2010) [79]	Ca/Si = 1.7~1.8	-	^35^Cl NMR, ^23^Na NMR	298	-	NVT	Coulombic, LJ	Chloride diffusion in C-S-H
Liu and Shi (2010) [80]	Tobermorite	LAMMPS	RDF	300	Vario-us	NVT	Custom potential (Buckingham, LJ)	Diffusion coefficient, structure at minimized energy
Dai and Hu (2011) [81]	Tobermorite 11 Å (Hamid’s)	Materials studio	XRD, RDF	300	-	NVT, NVE	Universal force field (UFF)	Distance between atomic and coordination number
Dai et al. (2011) [82]	Tobermorite (Hamid’s model)	Materials studio	-	300	-	NVT	COMPASS FF	Bulk modulus Shear Modulus, compressibility of C-S-H
Qomi et al. (2012) [83]	Tobermorite 14 Å and 11 Å	GULP	NMR	300	-	NPT, NVT	Custom potential (Buckingham, Coulomb, Morse, LJ)	Indentation modulus, Gibbs free energy, Young modulus
Fu et al. (2018) [84]	(CaO)_1.67_(SiO_2_)(H_2_O)_1.7_	LAMMPS	AFM, Nanoindentation	-	-	NPT	CLAYFF	Elastic modulus
Cao et al. (2020) [85]	Tobermorite 11 Å	Materials studio	NMR	298	-	NPT, NVT	Interatomic potential	Influence of pore size and fatigue loading
Sindu and Sasmal (2020) [86]	Tobermorite 11 Å	LAMMPS NAMD [87]		300	-	NPT, NVT	CSHFF	Behaviors of carbon nanotubes (CNT)-reinforced C-S-H

**Table 3:** Elastic properties of C-S-H gels computed by MD simulations.

C-S-H Model	Force Field	K (GPa)	G (GPa)	E (GPa)	Poisson’s Ratio
Tobermorite 9 Å	Others	53.36–86.25	26.72–37.44	72.38–112.72	0.23–0.35
[105,109,110]	CLAYFF	135.93	68.83	176.67	0.28
Tobermorite 11 Å	Others	38.45–77.19	17.91–40.42	46.5–103.25	0.27–0.33
[56,105,111]	CLAYFF	125.70	53.78	141.20	0.31
Tobermorite 14 Å	Others	20.7–56.42	15.33–31.65	41.47–80.00	0.24–0.35
[41,105,112,113,114]	CLAYFF	80.79	42.30	108.04	0.27
Clinotobermorite	Others	40.98–81.00	19.84–35.00	39.45–91.78	0.29–0.34
[105,109]	CLAYFF	104.12	47.59	123.89	0.3

**Table 4:** Mechanical properties of polymer-C-S-H nanocomposites. Reproduced from [181], with permission from Elsevier, 2019.

Property	C-S-H	C-S-H/PEG	C-S-H/PVA	C-S-H/PAA
Young’s modulus (GPa)	37.59	37.83	41.52	45.96
Tensile strength (GPa)	1.77	1.80	1.97	2.11
Failure strain	0.21	0.25	0.30	0.35

## References

[1] Scrivener K.L., Kirkpatrick R.J. (2008). Innovation in use and research on cementitious material. Cem. Concr. Res..

[2] Selvam R.P., Subramani V.J., Murray S., Hall K.D. (2009). Potential Application of Nanotechnology on Cement Based Materials.

[3] Bittnar Z., Bartos P.J., Nemecek J., Smilauer V., Zeman J. (2009). Nanotechnology in Construction: Proceedings of the NICOM3. Proceedings of the 3rd International Symposium on Nanotechnology in Construction.

[4] Jennings H.M., Bullard J.W., Thomas J.J., Andrade J.E., Chen J.J., Scherer G.W. (2008). Characterization and modeling of pores and surfaces in cement paste. J. Adv. Concr. Technol..

[5] Garboczi E., Bentz D. (1996). Modelling of the microstructure and transport properties of concrete. Constr. Build. Mater..

[6] Raki L., Beaudoin J., Alizadeh R. (2009). Nanotechnology applications for sustainable cement-based products. Nanotechnology in Construction 3.

[7] Scrivener K. (2009). Nanotechnology and cementitious materials. Nanotechnology in Construction 3.

[8] Garboczi E. (2009). Concrete nanoscience and nanotechnology: Definitions and applications. Nanotechnology in Construction 3.

[9] Sanchez F., Sobolev K. (2010). Nanotechnology in concrete—A review. Constr. Build. Mater..

[10] Papatzani S., Paine K., Calabria-Holley J. (2015). A comprehensive review of the models on the nanostructure of calcium silicate hydrates. Constr. Build. Mater..

[11] Odelius M., Bernasconi M., Parrinello M. (1997). Two dimensional ice adsorbed on mica surface. Phys. Rev. Lett..

[12] Marx D. (2004). Throwing tetrahedral dice. Science.

[13] Guillot B. (2002). A reappraisal of what we have learnt during three decades of computer simulations on water. J. Mol. Liq..

[14] Cygan R.T., Liang J.-J., Kalinichev A.G. (2004). Molecular models of hydroxide, oxyhydroxide, and clay phases and the development of a general force field. J. Phys. Chem. B.

[15] Du S., Wu J., AlShareedah O., Shi X. (2019). Nanotechnology in Cement-Based Materials: A Review of Durability, Modeling, and Advanced Characterization. Nanomaterials.

[16] Sha S., Wang M., Shi C., Xiao Y. (2020). Influence of the structures of polycarboxylate superplasticizer on its performance in cement-based materials-A review. Constr. Build. Mater..

[17] Vollpracht A., Lothenbach B., Snellings R., Haufe J. (2016). The pore solution of blended cements: A review. Mater. Struct..

[18] Taylor H.F. (1997). Cement Chemistry.

[19] Neville A.M. (1995). Properties of Concrete.

[20] Thomas J.J., Biernacki J.J., Bullard J.W., Bishnoi S., Dolado J.S., Scherer G.W., Luttge A. (2011). Modeling and simulation of cement hydration kinetics and microstructure development. Cem. Concr. Res..

[21] Bullard J.W., Jennings H.M., Livingston R.A., Nonat A., Scherer G.W., Schweitzer J.S., Scrivener K.L., Thomas J.J. (2011). Mechanisms of cement hydration. Cem. Concr. Res..

[22] Kurdowski W. (2014). Cement and Concrete Chemistry.

[23] Bensted J., Ghosh S.N. (1983). Hydration of Portland Cement, Advances in Cement Technology.

[24] Alizadeh R.A. (2009). Nanostructure and Engineering Properties of Basic and Modified Calcium-Silicate-Hydrate Systems. Doctoral Dissertation.

[25] Yang T. (2006). AFM Study of the Interactions between Moisture and the Surface of Cementitious Materials.

[26] Lothenbach B., Scrivener K., Hooton R. (2011). Supplementary cementitious materials. Cem. Concr. Res..

[27] Qomi M.A., Krakowiak K., Bauchy M., Stewart K., Shahsavari R., Jagannathan D., Brommer D.B., Baronnet A., Buehler M.J., Yip S. (2014). Combinatorial molecular optimization of cement hydrates. Nat. Commun..

[28] Taylor H.W. (1950). 726. Hydrated calcium silicates. Part I. Compound formation at ordinary temperatures. J. Chem. Soc. (Resumed.).

[29] Nonat A., Lecoq X. (1998). The Structure, Stoichiometry and Properties of CSH Prepared by C_3_S Hydration Under Controlled Condition. Nuclear Magnetic Resonance Spectroscopy of Cement-Based Materials.

[30] Fonseca P., Jennings H.M. (2010). The effect of drying on early-age morphology of C–S–H as observed in environmental SEM. Cem. Concr. Res..

[31] Powers T.C., Brownyard T.L. (1946). Studies of the physical properties of hardened Portland cement paste. Journal Proceedings.

[32] Powers T.C. (1958). Structure and physical properties of hardened Portland cement paste. J. Am. Ceram. Soc..

[33] Taplin J. (1959). A method for following the hydration reaction in portland cement paste. Aust. J. Appl. Sci..

[34] Diamond S. (1976). Cement Paste Microstructure—An Overview at Several Levels. Hydtaulic Cement Paste—Their Structure and Properties.

[35] Aquino R.J. (2012). Conceptual modelling of CSH formation and the occurrence of Hadley grains in hardened cement paste. Proceedings of the 9th fib International PhD Symposium in Civil Engineering.

[36] Richardson I.G. (1999). The nature of CSH in hardened cements. Cem. Concr. Res..

[37] Tennis P.D., Jennings H.M. (2000). A model for two types of calcium silicate hydrate in the microstructure of Portland cement pastes. Cem. Concr. Res..

[38] Jennings H.M. (2004). Colloid model of C–S–H and implications to the problem of creep and shrinkage. Mater. Struct..

[39] Jennings H.M. (2008). Refinements to colloid model of CSH in cement: CM-II. Cem. Concr. Res..

[40] Nicoleau L. (2010). New calcium silicate hydrate network. Transp. Res. Rec..

[41] Pellenq R.-M., Lequeux N., Van Damme H. (2008). Engineering the bonding scheme in C–S–H: The iono-covalent framework. Cem. Concr. Res..

[42] Pelisser F., Gleize P.J.P., Mikowski A. (2012). Effect of the Ca/Si molar ratio on the micro/nanomechanical properties of synthetic CSH measured by nanoindentation. J. Phys. Chem. C.

[43] Richardson I., Groves G. (1993). Microstructure and microanalysis of hardened ordinary Portland cement pastes. J. Mater. Sci..

[44] Richardson I. (2000). The nature of the hydration products in hardened cement pastes. Cem. Concr. Compos..

[45] Taylor H.F. (1986). Proposed structure for calcium silicate hydrate gel. J. Am. Ceram. Soc..

[46] Wieker W., Grimmer A.-R., Winkler A., Mägi M., Tarmak M., Lippmaa E. (1982). Solid-state high-resolution 29Si NMR spectroscopy of synthetic 14 Å, 11 Å and 9 Å tobermorites. Cem. Concr. Res..

[47] Komarneni S., Roy D., Fyfe C., Kennedy G. (1987). Naturally occurring 1.4 nm tobermorite and synthetic jennite: Characterization by 27Al and 29Si MASNMR spectroscopy and cation exchange properties. Cem. Concr. Res..

[48] Hamid S. (1981). The crystal structure of the 11 Ä natural tobermorite Ca_2.25_[Si_3_O_7.5_(OH)_1.5_]·1H_2_O. Z. Für Krist. Cryst. Mater..

[49] Hoffmann C., Armbruster T. (1997). Clinotobermorite, Ca_5_[Si_3_0_8_(OH)]_2_ 4H_2_0-Ca_5_[Si_6_Oi_7_] 5H_2_0, CSH (I) type cement mineral: Determination of the substructure. Z. Fur Krist..

[50] Bonaccorsi E., Merlino S., Taylor H. (2004). The crystal structure of jennite, Ca_9_Si_6_O_18_(OH)_6_ 8H_2_O. Cem. Concr. Res..

[51] Carpenter A., Chalmers R., Gard J., Speakman K., Taylor H. (1966). Jennite, a new mineral. Am. Mineral. J. Earth Planet. Mater..

[52] Taylor H.F.W. (1993). Nanostructure of CSH: Current status. Adv. Cem. Based Mater..

[53] Cong X., Kirkpatrick R.J. (1996). 29Si MAS NMR study of the structure of calcium silicate hydrate. Adv. Cem. Based Mater..

[54] Richardson I. (2004). Tobermorite/jennite-and tobermorite/calcium hydroxide-based models for the structure of CSH: Applicability to hardened pastes of tricalcium silicate, β-dicalcium silicate, Portland cement, and blends of Portland cement with blast-furnace slag, metakaolin, or silica fume. Cem. Concr. Res..

[55] Shahsavari R., Buehler M.J., Pellenq R.J.M., Ulm F.J. (2009). First-principles study of elastic constants and interlayer interactions of complex hydrated oxides: Case study of tobermorite and jennite. J. Am. Ceram. Soc..

[56] Shahsavari R., Pellenq R.J.-M., Ulm F.-J. (2011). Empirical force fields for complex hydrated calcio-silicate layered materials. Phys. Chem. Chem. Phys..

[57] Merlino S., Bonaccorsi E., Armbruster T. (2001). The real structure of tobermorite 11 Å: Normal and anomalous forms, OD character and polytypic modifications. Eur. J. Mineral..

[58] Faucon P., Delaye J., Virlet J., Jacquinot J., Adenot F. (1997). Study of the structural properties of the C–S–H (I) BY molecular dynamics simulation. Cem. Concr. Res..

[59] Allen A.J., Thomas J.J., Jennings H.M. (2007). Composition and density of nanoscale calcium–silicate–hydrate in cement. Nat. Mater..

[60] Chen J.J., Thomas J.J., Taylor H.F., Jennings H.M. (2004). Solubility and structure of calcium silicate hydrate. Cem. Concr. Res..

[61] Kirkpatrick R.J., Yarger J., McMillan P.F., Ping Y., Cong X. (1997). Raman spectroscopy of CSH, tobermorite, and jennite. Adv. Cem. Based Mater..

[62] Cong X., Kirkpatrick R.J. (1996). 29Si and 17O NMR investigation of the structure of some crystalline calcium silicate hydrates. Adv. Cem. Based Mater..

[63] Bonaccorsi E., Merlino S., Kampf A.R. (2005). The crystal structure of tobermorite 14 Å (plombierite), a C–S–H phase. J. Am. Ceram. Soc..

[64] Jennings H.M., Thomas J.J., Gevrenov J.S., Constantinides G., Ulm F.-J. (2007). A multi-technique investigation of the nanoporosity of cement paste. Cem. Concr. Res..

[65] Constantinides G., Ulm F.-J. (2004). The effect of two types of CSH on the elasticity of cement-based materials: Results from nanoindentation and micromechanical modeling. Cem. Concr. Res..

[66] Jennings H.M. (2000). A model for the microstructure of calcium silicate hydrate in cement paste. Cem. Concr. Res..

[67] Dai Y., Post J.E. (1995). Crystal structure of hillebrandite: A natural analogue of calcium silicate hydrate (CSH) phases in Portland cement. Am. Mineral..

[68] Fu J., Bernard F., Kamali-Bernard S. (2018). Assessment of the elastic properties of amorphous calcium silicates hydrates (I) and (II) structures by molecular dynamics simulation. Mol. Simul..

[69] Pellenq R.J.-M., Kushima A., Shahsavari R., Van Vliet K.J., Buehler M.J., Yip S., Ulm F.-J. (2009). A realistic molecular model of cement hydrates. Proc. Natl. Acad. Sci. USA.

[70] Merlin F., Lombois H., Joly S., Lequeux N., Halary J.-L., Van Damme H. (2002). Cement-polymer and clay-polymer nano-and meso-composites: Spotting the difference. J. Mater. Chem..

[71] Buehler M.J., van Duin A.C., Goddard III W.A. (2006). Multiparadigm modeling of dynamical crack propagation in silicon using a reactive force field. Phys. Rev. Lett..

[72] Manzano H., Moeini S., Marinelli F., Van Duin A.C., Ulm F.-J., Pellenq R.J.-M. (2012). Confined water dissociation in microporous defective silicates: Mechanism, dipole distribution, and impact on substrate properties. J. Am. Chem. Soc..

[73] Van Duin A.C., Dasgupta S., Lorant F., Goddard W.A. (2001). ReaxFF: A reactive force field for hydrocarbons. J. Phys. Chem. A.

[74] Hou D. (2020). The Future and Development Trends of Computational Chemistry Applied in Concrete Science. Molecular Simulation on Cement-Based Materials.

[75] Bonnaud P., Ji Q., Coasne B., Pellenq R.-M., Van Vliet K. (2012). Thermodynamics of water confined in porous calcium-silicate-hydrates. Langmuir.

[76] Dolado J.S., Griebel M., Hamaekers J. (2007). A molecular dynamic study of cementitious calcium silicate hydrate (C–S–H) gels. J. Am. Ceram. Soc..

[77] Kalinichev A.G., Wang J., Kirkpatrick R.J. (2007). Molecular dynamics modeling of the structure, dynamics and energetics of mineral–water interfaces: Application to cement materials. Cem. Concr. Res..

[78] Murray S.J., Subramani V.J., Selvam R.P., Hall K.D. (2010). Molecular dynamics to understand the mechanical behavior of cement paste. Transp. Res. Rec..

[79] Pan T., Xia K., Wang L. (2010). Chloride binding to calcium silicate hydrates (CSH) in cement paste: A molecular dynamics analysis. Int. J. Pavement Eng..

[80] Liu Y., Shi X. (2010). Molecular dynamics study of interaction between corrosion inhibitors, nanoparticles, and other minerals in hydrated cement. Transp. Res. Rec..

[81] Hu P., Dai W. Study on Molecular Dynamics Simulation of Calcium Silicate Hydrate (CSH) Gels. Proceedings of International Conference on Intelligent Computing and Information Science.

[82] Dai W., Shui Z., Duan P. (2011). Molecular dynamics simulation on calcium silicate hydrate doped organic molecules. Proceedings of International Conference on Intelligent Computing and Information Science.

[83] Abdolhosseini Qomi M.J., Ulm F.J., Pellenq R.J.M. (2012). Evidence on the dual nature of aluminum in the calcium-silicate-hydrates based on atomistic simulations. J. Am. Ceram. Soc..

[84] Fu J., Kamali-Bernard S., Bernard F., Cornen M. (2018). Comparison of mechanical properties of CSH and portlandite between nano-indentation experiments and a modeling approach using various simulation techniques. Compos. Part B Eng..

[85] Cao Q., Xu Y., Fang J., Song Y., Wang Y., You W. (2020). Influence of Pore Size and Fatigue Loading on NaCl Transport Properties in CSH Nanopores: A Molecular Dynamics Simulation. Materials.

[86] Sindu B., Sasmal S. (2020). Molecular dynamics simulations for evaluation of surfactant compatibility and mechanical characteristics of carbon nanotubes incorporated cementitious composite. Constr. Build. Mater..

[87] Phillips J.C., Braun R., Wang W., Gumbart J., Tajkhorshid E., Villa E., Chipot C., Skeel R.D., Kale L., Schulten K. (2005). Scalable molecular dynamics with NAMD. J. Comput. Chem..

[88] Bordallo H.N., Aldridge L.P., Desmedt A. (2006). Water dynamics in hardened ordinary portland cement paste or concrete: From quasielastic neutron scattering. J. Phys. Chem. B.

[89] Packer K.J. (1977). The dynamics of water in heterogeneous systems. Philos. Trans. R. Soc. Lond. B Biol. Sci..

[90] Israelachvili J.N., Pashley R.M. (1983). Molecular layering of water at surfaces and origin of repulsive hydration forces. Nature.

[91] Israelachvili J., Wennerström H. (1996). Role of hydration and water structure in biological and colloidal interactions. Nature.

[92] Kalinichev A.G., Kirkpatrick R.J., Cygan R.T. (2000). Molecular modeling of the structure and dynamics of the interlayer and surface species of mixed-metal layered hydroxides: Chloride and water in hydrocalumite (Friedel’s salt). Am. Mineral..

[93] Fenter P., Geissbühler P., DiMasi E., Srajer G., Sorensen L., Sturchio N. (2000). Surface speciation of calcite observed in situ by high-resolution X-ray reflectivity. Geochim. Cosmochim. Acta.

[94] Raviv U., Laurat P., Klein J. (2001). Fluidity of water confined to subnanometre films. Nature.

[95] Brown G.E. (2001). How minerals react with water. Science.

[96] Sakuma H., Tsuchiya T., Kawamura K., Otsuki K. (2003). Large self-diffusion of water on brucite surface by ab initio potential energy surface and molecular dynamics simulations. Surf. Sci..

[97] Hou D., Li Z., Zhao T. (2015). Reactive force field simulation on polymerization and hydrolytic reactions in calcium aluminate silicate hydrate (C–A–S–H) gel: Structure, dynamics and mechanical properties. Rsc Adv..

[98] Hou D., Li Z. (2015). Large-scale simulation of calcium silicate hydrate by molecular dynamics. Adv. Cem. Res..

[99] Hou D., Zhu Y., Lu Y., Li Z. (2014). Mechanical properties of calcium silicate hydrate (C–S–H) at nano-scale: A molecular dynamics study. Mater. Chem. Phys..

[100] Hou D., Zhao T., Wang P., Li Z., Zhang J. (2014). Molecular dynamics study on the mode I fracture of calcium silicate hydrate under tensile loading. Eng. Fract. Mech..

[101] Hou D., Ma H., Zhu Y., Li Z. (2014). Calcium silicate hydrate from dry to saturated state: Structure, dynamics and mechanical properties. Acta Mater..

[102] Major R., Houston J., McGrath M., Siepmann J., Zhu X.-Y. (2006). Viscous water meniscus under nanoconfinement. Phys. Rev. Lett..

[103] Youssef M., Pellenq R.J.-M., Yildiz B. (2011). Glassy nature of water in an ultraconfining disordered material: The case of calcium–silicate–hydrate. J. Am. Chem. Soc..

[104] Manzano H., Dolado J., Guerrero A., Ayuela A. (2007). Mechanical properties of crystalline calcium-silicate-hydrates: Comparison with cementitious C-S-H gels. Phys. Status Solidi.

[105] Tarighat A., Tavakoli D. (2019). Estimation of the elastic properties of important calcium silicate hydrates in nano scale—A molecular dynamics approach. J. Rehabil. Civ. Eng..

[106] Nonat A. (2000). PRO 13: 2nd International RILEM Symposium on Hydration and Setting—Why Does Cement Set? An Interdisciplinary Approach.

[107] Acker P. (2004). Swelling, shrinkage and creep: A mechanical approach to cement hydration. Mater. Struct..

[108] Ulm F.-J., Constantinides G., Heukamp F.H. (2004). Is concrete a poromechanics materials?—A multiscale investigation of poroelastic properties. Mater. Struct..

[109] Manzano Moro H. (2009). Atomistic Simulation Studies of the Cement Paste Components.

[110] Hajilar S., Shafei B. (2015). Nano-scale investigation of elastic properties of hydrated cement paste constituents using molecular dynamics simulations. Comput. Mater. Sci..

[111] Dharmawardhana C., Misra A., Aryal S., Rulis P., Ching W. (2013). Role of interatomic bonding in the mechanical anisotropy and interlayer cohesion of CSH crystals. Cem. Concr. Res..

[112] Vandamme M., Ulm F.-J., Fonollosa P. (2010). Nanogranular packing of C–S–H at substochiometric conditions. Cem. Concr. Res..

[113] Richardson I., Groves G. (1992). Models for the composition and structure of calcium silicate hydrate (C–S–H) gel in hardened tricalcium silicate pastes. Cem. Concr. Res..

[114] Al-Ostaz A., Wu W., Cheng A.-D., Song C. (2010). A molecular dynamics and microporomechanics study on the mechanical properties of major constituents of hydrated cement. Compos. Part B Eng..

[115] Larcher M. (2009). Development of discrete cracks in concrete loaded by shock waves. Int. J. Impact Eng..

[116] Maurel O., Reess T., Matallah M., De Ferron A., Chen W., La Borderie C., Pijaudier-Cabot G., Jacques A., Rey-Bethbeder F. (2010). Electrohydraulic shock wave generation as a means to increase intrinsic permeability of mortar. Cem. Concr. Res..

[117] Yan D., Lin G. (2006). Dynamic properties of concrete in direct tension. Cem. Concr. Res..

[118] Lin W., Zhang C., Fu J., Xin H. (2018). Dynamic mechanical behaviors of calcium silicate hydrate under shock compression loading using molecular dynamics simulation. J. Non Cryst. Solids.

[119] Camacho-Ballesta C., Galao Ó., Baeza F.J., Zornoza E., Garcés P. (2019). Durability and Mechanical Properties of CNT Cement Composites. Service Life and Durability of Reinforced Concrete Structures.

[120] Zhu X., Gao Y., Dai Z., Corr D.J., Shah S.P. (2018). Effect of interfacial transition zone on the Young’s modulus of carbon nanofiber reinforced cement concrete. Cem. Concr. Res..

[121] Alharbi Y., Cho B.H., An J., Nam B.H. (2020). Rheological Behaviors of Edge-Oxidized Graphene Oxide Cement Composites. J. Mater. Civ. Eng..

[122] Alharbi Y., An J., Cho B.H., Khawaji M., Chung W., Nam B.H. (2018). Mechanical and sorptivity characteristics of edge-oxidized graphene oxide (EOGO)-cement composites: Dry- and wet-mix design methods. Nanomaterials.

[123] Metaxa Z.S., Konsta-Gdoutos M.S., Shah S.P. (2010). Carbon nanofiber–reinforced cement-based materials. Transp. Res. Rec..

[124] Kis A., Csanyi G., Salvetat J.-P., Lee T.-N., Couteau E., Kulik A., Benoit W., Brugger J., Forro L. (2004). Reinforcement of single-walled carbon nanotube bundles by intertube bridging. Nat. Mater..

[125] Demczyk B.G., Wang Y.M., Cumings J., Hetman M., Han W., Zettl A., Ritchie R. (2002). Direct mechanical measurement of the tensile strength and elastic modulus of multiwalled carbon nanotubes. Mater. Sci. Eng. A.

[126] Lee C., Wei X., Kysar J.W., Hone J. (2008). Measurement of the elastic properties and intrinsic strength of monolayer graphene. Science.

[127] Meng W., Khayat K.H. (2016). Mechanical properties of ultra-high-performance concrete enhanced with graphite nanoplatelets and carbon nanofibers. Compos. Part B Eng..

[128] Cwirzen A., Habermehl-Cwirzen K., Penttala V. (2008). Surface decoration of carbon nanotubes and mechanical properties of cement/carbon nanotube composites. Adv. Cem. Res..

[129] Musso S., Tulliani J.-M., Ferro G., Tagliaferro A. (2009). Influence of carbon nanotubes structure on the mechanical behavior of cement composites. Compos. Sci. Technol..

[130] Makar J.M., Chan G.W. (2009). Growth of cement hydration products on single-walled carbon nanotubes. J. Am. Ceram. Soc..

[131] Harris P.J. (2004). Carbon nanotube composites. Int. Mater. Rev..

[132] Raki L., Beaudoin J., Alizadeh R., Makar J., Sato T. (2010). Cement and concrete nanoscience and nanotechnology. Materials.

[133] Konsta-Gdoutos M.S., Metaxa Z.S., Shah S.P. (2010). Highly dispersed carbon nanotube reinforced cement based materials. Cem. Concr. Res..

[134] Eftekhari M., Mohammadi S. (2016). Multiscale dynamic fracture behavior of the carbon nanotube reinforced concrete under impact loading. Int. J. Impact Eng..

[135] Eftekhari M., Mohammadi S., Khoei A.R. (2013). Effect of defects on the local shell buckling and post-buckling behavior of single and multi-walled carbon nanotubes. Comput. Mater. Sci..

[136] Manzur T. (2011). Nano-Modified Cement Composites and Its Applicability as Concrete Repair Material. Ph.D. Thesis.

[137] Li G.Y., Wang P.M., Zhao X. (2005). Mechanical behavior and microstructure of cement composites incorporating surface-treated multi-walled carbon nanotubes. Carbon.

[138] Kuila T., Bose S., Mishra A.K., Khanra P., Kim N.H., Lee J.H. (2012). Chemical functionalization of graphene and its applications. Prog. Mater. Sci..

[139] Stankovich S., Dikin D.A., Piner R.D., Kohlhaas K.A., Kleinhammes A., Jia Y., Wu Y., Nguyen S.T., Ruoff R.S. (2007). Synthesis of graphene-based nanosheets via chemical reduction of exfoliated graphite oxide. Carbon.

[140] Kuilla T., Bhadra S., Yao D., Kim N.H., Bose S., Lee J.H. (2010). Recent advances in graphene based polymer composites. Prog. Polym. Sci..

[141] Alkhateb H., Al-Ostaz A., Cheng A.H.-D., Li X. (2013). Materials genome for graphene-cement nanocomposites. J. Nanomechanics Micromechanics.

[142] Chuah S., Pan Z., Sanjayan J.G., Wang C.M., Duan W.H. (2014). Nano reinforced cement and concrete composites and new perspective from graphene oxide. Constr. Build. Mater..

[143] Ghazizadeh S., Duffour P., Skipper N.T., Bai Y. (2018). Understanding the behaviour of graphene oxide in Portland cement paste. Cem. Concr. Res..

[144] Lv S., Liu J., Sun T., Ma Y., Zhou Q. (2014). Effect of GO nanosheets on shapes of cement hydration crystals and their formation process. Constr. Build. Mater..

[145] Gong K., Pan Z., Korayem A.H., Qiu L., Li D., Collins F., Wang C.M., Duan W.H. (2015). Reinforcing effects of graphene oxide on portland cement paste. J. Mater. Civ. Eng..

[146] Babak F., Abolfazl H., Alimorad R., Parviz G. (2014). Preparation and mechanical properties of graphene oxide: Cement nanocomposites. Sci. World J..

[147] Devasena M., Karthikeyan J. (2015). Investigation on strength properties of graphene oxide concrete. Int. J. Eng. Sci. Invent. Res. Dev..

[148] Li W., Li X., Chen S.J., Long G., Liu Y.M., Duan W.H. (2017). Effects of nanoalumina and graphene oxide on early-age hydration and mechanical properties of cement paste. J. Mater. Civ. Eng..

[149] Li X., Liu Y.M., Li W.G., Li C.Y., Sanjayan J.G., Duan W.H., Li Z. (2017). Effects of graphene oxide agglomerates on workability, hydration, microstructure and compressive strength of cement paste. Constr. Build. Mater..

[150] Li X., Lu Z., Chuah S., Li W., Liu Y., Duan W.H., Li Z. (2017). Effects of graphene oxide aggregates on hydration degree, sorptivity, and tensile splitting strength of cement paste. Compos. Part A Appl. Sci. Manuf..

[151] Lu L., Ouyang D. (2017). Properties of cement mortar and ultra-high strength concrete incorporating graphene oxide nanosheets. Nanomaterials.

[152] Mohammed A., Sanjayan J., Nazari A., Al-Saadi N. (2017). Effects of graphene oxide in enhancing the performance of concrete exposed to high-temperature. Aust. J. Civ. Eng..

[153] Mokhtar M., Abo-El-Enein S., Hassaan M., Morsy M., Khalil M. (2017). Mechanical performance, pore structure and micro-structural characteristics of graphene oxide nano platelets reinforced cement. Constr. Build. Mater..

[154] Sharma S., Kothiyal N. (2015). Influence of graphene oxide as dispersed phase in cement mortar matrix in defining the crystal patterns of cement hydrates and its effect on mechanical, microstructural and crystallization properties. RSC Adv..

[155] Yang H., Monasterio M., Cui H., Han N. (2017). Experimental study of the effects of graphene oxide on microstructure and properties of cement paste composite. Compos. Part A Appl. Sci. Manuf..

[156] Lv S., Ma Y., Qiu C., Sun T., Liu J., Zhou Q. (2013). Effect of graphene oxide nanosheets of microstructure and mechanical properties of cement composites. J. Constr. Build. Mater..

[157] Pan Z., He L., Qiu L., Korayem A.H., Li G., Zhu J.W., Collins F., Li D., Duan W.H., Wang M.C. (2015). Mechanical properties and microstructure of a graphene oxide–cement composite. Cem. Concr. Compos..

[158] Le J.-L., Du H., Dai Pang S. (2014). Use of 2D Graphene Nanoplatelets (GNP) in cement composites for structural health evaluation. Compos. Part B Eng..

[159] Ranjbar N., Mehrali M., Mehrali M., Alengaram U.J., Jumaat M.Z. (2015). Graphene nanoplatelet-fly ash based geopolymer composites. Cem. Concr. Res..

[160] Horszczaruk E., Mijowska E., Kalenczuk R.J., Aleksandrzak M., Mijowska S. (2015). Nanocomposite of cement/graphene oxide—Impact on hydration kinetics and Young’s modulus. Constr. Build. Mater..

[161] Saafi M., Tang L., Fung J., Rahman M., Liggat J. (2015). Enhanced properties of graphene/fly ash geopolymeric composite cement. Cem. Concr. Res..

[162] Mohammed A., Sanjayan J.G., Duan W., Nazari A. (2015). Incorporating graphene oxide in cement composites: A study of transport properties. Constr. Build. Mater..

[163] Shang Y., Zhang D., Yang C., Liu Y., Liu Y. (2015). Effect of graphene oxide on the rheological properties of cement pastes. Constr. Build. Mater..

[164] Garmor Technology. https://garmortech.com/#technology.

[165] Cho B.H., Khawaji M., Nam B.H., Alharbi Y., An J. (2019). Static and Cyclic Flexural Behaviors of Edge-Oxidized Graphene Oxide Cement Composites. J. Mater. Civ. Eng..

[166] Cho B.H., Khawaji M., Alharbi Y., An J., Nam B.H. Effects of Edge-Oxidized Graphene Oxide (EOGO) on Flexural Fatigue Behaviors of Cement Mortar. Proceedings of the Transportation Research Board 98th Annual Meeting.

[167] An J., Cho B.H., Alharbi Y., Khawaji M., McInnis M., Nam B.H. Optimized mix design for graphene oxide nanoflake (GONF)–cement composite. Proceedings of the Transportation Research Board 97th Annual Meeting.

[168] Khawaji M., Cho B.H., Nam B.H., Alharbi Y., An J. (2020). Edge-Oxidized Graphene Oxide as Additive in Fiber-Reinforced Concrete: Effects on Fresh and Hardened Properties. J. Mater. Civ. Eng..

[169] Khawaji M., Cho B.H., Alharbi Y., An J., Nam B.H. Effects of Edge-Oxidized Graphene Oxide (EOGO) on Workability and Mechanical Strength of Steel Fiber Reinforced Concrete. Proceedings of the Transportation Research Board 98th Annual Meeting.

[170] Eftekhari M., Mohammadi S. (2016). Molecular dynamics simulation of the nonlinear behavior of the CNT-reinforced calcium silicate hydrate (C–S–H) composite. Int. J. Impact Eng..

[171] Sanchez F., Zhang L. (2008). Molecular dynamics modeling of the interface between surface functionalized graphitic structures and calcium–silicate–hydrate: Interaction energies, structure, and dynamics. J. Colloid Interface Sci..

[172] Al-Muhit B., Sanchez F. (2020). Nano-engineering of the mechanical properties of tobermorite 14 Å with graphene via molecular dynamics simulations. Constr. Build. Mater..

[173] Hou D., Lu Z., Li X., Ma H., Li Z. (2017). Reactive molecular dynamics and experimental study of graphene-cement composites: Structure, dynamics and reinforcement mechanisms. Carbon.

[174] Kai M., Zhang L., Liew K. (2019). Graphene and graphene oxide in calcium silicate hydrates: Chemical reactions, mechanical behaviors and interfacial sliding. Carbon.

[175] Hou D., Yang T., Tang J., Li S. (2018). Reactive force-field molecular dynamics study on graphene oxide reinforced cement composite: Functional group de-protonation, interfacial bonding and strengthening mechanism. Phys. Chem. Chem. Phys..

[176] Yang T., Jia Y., Hou D., Li H., Jiang J., Zhang J. (2018). Molecular dynamics study on the weakening effect of moisture content on graphene oxide reinforced cement composite. Chem. Phys. Lett..

[177] Han F., Lin X., Zhang C. (2015). Effects of CMC polymer on mechanical properties of cement mortars. J. Build. Sci..

[178] Wang Z. (2014). Performance of polymer cement concrete. J. Transp. Stand..

[179] Guo X.H., Zhang X.L., Cao H. (2014). Experimental investigation on impact ductility of polymer-modified concrete subjected to falling weight loading. Adv. Mater. Res..

[180] Xu F., Zhu J., Chen J., Zhou M., Liu H. (2012). Study on the Interfacial Adhesive Performance and Enhancement Mechanism of Polymer Modified Cement Paste Interface Agent. Mater. Rev..

[181] Hou D., Yu J., Wang P. (2019). Molecular dynamics modeling of the structure, dynamics, energetics and mechanical properties of cement-polymer nanocomposite. Compos. Part B Eng..

[182] Wang L., Tian Z., Ma G., Zhang M. (2020). Interlayer bonding improvement of 3D printed concrete with polymer modified mortar: Experiments and molecular dynamics studies. Cem. Concr. Compos..

[183] Hosseini E., Zakertabrizi M., Korayem A.H., Xu G. (2019). A novel method to enhance the interlayer bonding of 3D printing concrete: An experimental and computational investigation. Cem. Concr. Compos..

[184] Zhou Y., Hou D., Jiang J., Liu L., She W., Yu J. (2018). Experimental and molecular dynamics studies on the transport and adsorption of chloride ions in the nano-pores of calcium silicate phase: The influence of calcium to silicate ratios. Microporous Mesoporous Mater..

